# Novel *IFT122* mutation associated with impaired ciliogenesis and cranioectodermal dysplasia

**DOI:** 10.1002/mgg3.44

**Published:** 2013-12-10

**Authors:** Anas M Alazami, Mohammed Zain Seidahmed, Fatema Alzahrani, Adam O Mohammed, Fowzan S Alkuraya

**Affiliations:** 1 Department of Genetics, King Faisal Specialist Hospital and Research CenterRiyadh, Saudi Arabia; 2 Department of Pediatrics, Security Forces HospitalRiyadh, Saudi Arabia; 3 Department of Pediatrics, National Guard HospitalDammam, Saudi Arabia; 4 Department of Anatomy and Cell Biology, College of Medicine, Alfaisal UniversityRiyadh, Saudi Arabia

**Keywords:** Ciliopathy, craniosynostosis, intraflagellar transport

## Abstract

Cranioectodermal dysplasia (CED) is a very rare autosomal recessive disorder characterized by a recognizable craniofacial profile in addition to ectodermal manifestations involving the skin, hair, and teeth. Four genes are known to be mutated in this disorder, all involved in the ciliary intraflagellar transport confirming that CED is a ciliopathy. In a multiplex consanguineous family with typical CED features in addition to intellectual disability and severe cutis laxa, we used autozygosity-guided candidate gene analysis to identify a novel homozygous mutation in *IFT122*, and demonstrated impaired ciliogenesis in patient fibroblasts. This report on *IFT122* broadens the phenotype of CED and expands its allelic heterogeneity.

## Brief Report

Cranioectodermal dysplasia (CED) is a skeletal dysplasia characterized by typical craniofacial features in the form of dolichocephaly, sagittal craniosynostosis, and facial dysmorphism (frontal bossing, epicanthic folds, flat nose with anteverted nares, and everted lower lip), and skeletal anomalies in the form of narrow thorax and short extremities, in addition to ectodermal dysplastic features in the form of thin sparse scalp hair and micro/hypodontia (Levin et al. 1977). Since its first description in 1975 (Sensenbrenner et al. 1975), less than 50 cases have been reported indicating the rarity of this syndrome. The subsequent expansion of the phenotype to include corpus callosal dysgenesis, hepatic fibrosis, nephrophthisis, and retinitis pigmentosa made it likely that CED is a ciliopathy (Konstantinidou et al. 2009). Indeed, genetic studies confirmed this hypothesis by identifying mutations in four genes all encoding components of the ciliary intraflagellar transport complex-A (*IFT122*,*WDR35*,*C14ORF179*, and *WDR19*) (Gilissen et al. 2010; Walczak-Sztulpa et al. 2010; Arts et al. 2011; Bredrup et al. 2011). Not unlike other ciliopathy disease genes, mutations in some of these genes have been observed to cause overlapping ciliopathy phenotypes such as the finding of *WDR35* mutations in short rib-polydactyly syndrome (Mill et al. 2011). Thus, additional reports of mutations in these genes will be critical to our understanding of the spectrum of resulting phenotypes.

**Figure 1:**
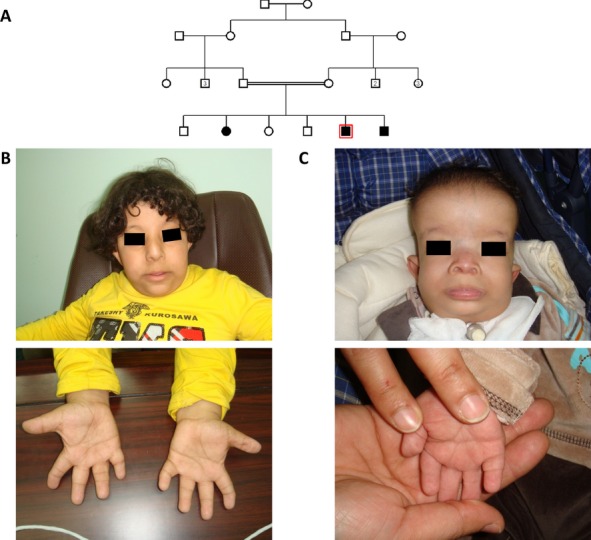
The family reported in this study. (A) Pedigree of the family with the index case boxed in red. Facial and hand profiles for the two male patients are given in (B) and (C). Note the typical facial features (prominent forehead, depressed nasal bridge, anteverted nares, and everted lower lip) and typical hand features (brachdactyly, single interphalangeal crease for some fingers and clinodactyly). Please note the redundant palm skin in the lower panels, consistent with cutis laxa.

**Figure 2:**
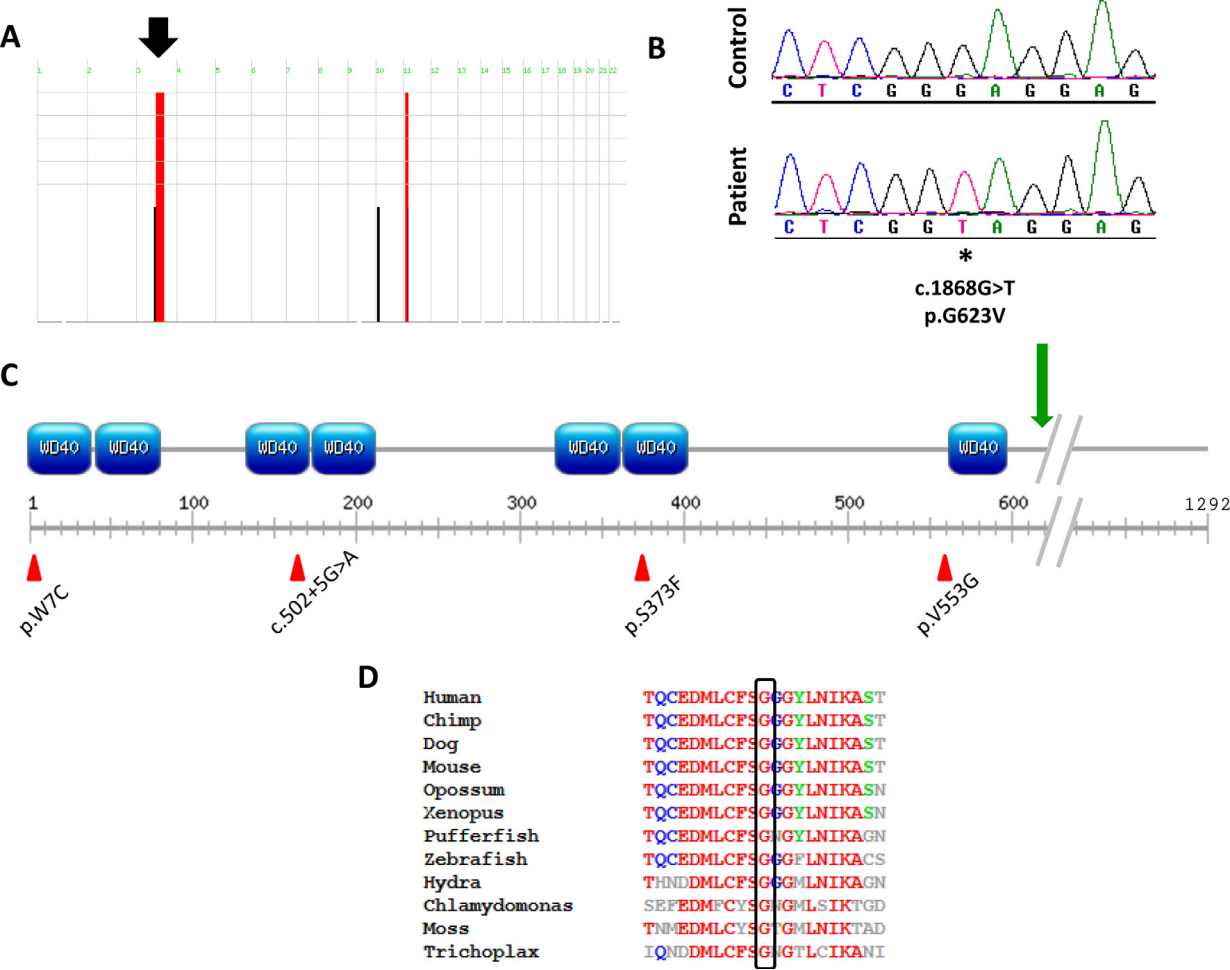
A missense mutation in *IFT122* causes cranioectodermal dysplasia in the reported family. (A) Homozygosity Mapper reveals two regions of autozygosity which are shared by the three patients genomewide. The *IFT122* locus is indicated with an arrow. (B) Sequence chromatogram of one control individual and one patient, with the site of mutation denoted by an asterisk. (C) Schematic of the IFT122 protein indicating the position of WD40 domains. The mutation reported here is located with a green arrow, while all previously published mutations are given below the schematic (red arrowheads). (D) Protein alignment data reveal that the affected amino acid residue is highly conserved across species, down to moss and trichoplax.

In this report, we describe a multiplex family containing three affected siblings born to healthy first cousin Saudi parents (Fig. 1A). In addition to the classical features of CED (Table 1, Fig. 1B and C), they all had markedly lax skin with joint laxity fulfilling the clinical definition of cutis laxa, but none had evidence of retinal involvement and only the oldest patient developed end-stage renal failure. In addition, the two older siblings have confirmed intellectual disability with intelligence quotient of 70, a feature that has not been reported in CED. Given the consanguinity of the parents and the genetic heterogeneity of CED, we pursued autozygome-guided candidate gene sequencing essentially as described before (Alkuraya 2012). The autozygome of the three siblings overlapped on just two genomic regions (chr3:102718000-136160000, and chr11:13395000-27766000, hg19 build) (Fig. 2A). *IFT122* was the only known CED disease gene mapping to either of the two regions. Direct sequencing of *IFT122* revealed a novel homozygous missense mutation (c.1868G>T, p.G623V; NM_052985.2) (Fig. 2B). The affected amino acid residue is highly conserved across species (Fig. 2D), is associated with high *in silico* pathogenicity scores (PolyPhen 1.0 and SIFT 0.0) and was absent from 374 Saudi control chromosomes.

**Table 1:** Clinical features.

Clinical feature	Patient 1	Patient 2	Patient 3
Age	22 years	9 years	2 years
Frequent chest infection	Female	Male	Male
Sagittal craniosynostosis	+	+	+
Dolichocephaly	+	−	+
Epicanthal folds	+	+	+
Broad nasal bridge	+	+	+
Anteverted nares	+	+	+
Everted lower lip	−	−	−
Micro/Hypodontia	+	+	+
Narrow thorax	+	−	+
Short limbs	+	+	+
Brachydactyly	+	+	+
Clinodactyly	+	+	+
Joint hypermobility	+	+	+
Cutis laxa	+	+	+
Osteoporosis	?	−	−
Retinal dystrophy	−	−	−
Nephronophthisis	ESRF	−	−
Congenital heart disease	−	−	−
Frequent chest infection	+	+	+
Others	ID	ID	−

ESRF, end stage renal failure; ID, intellectual disability; ?, unknown.

**Figure 3:**
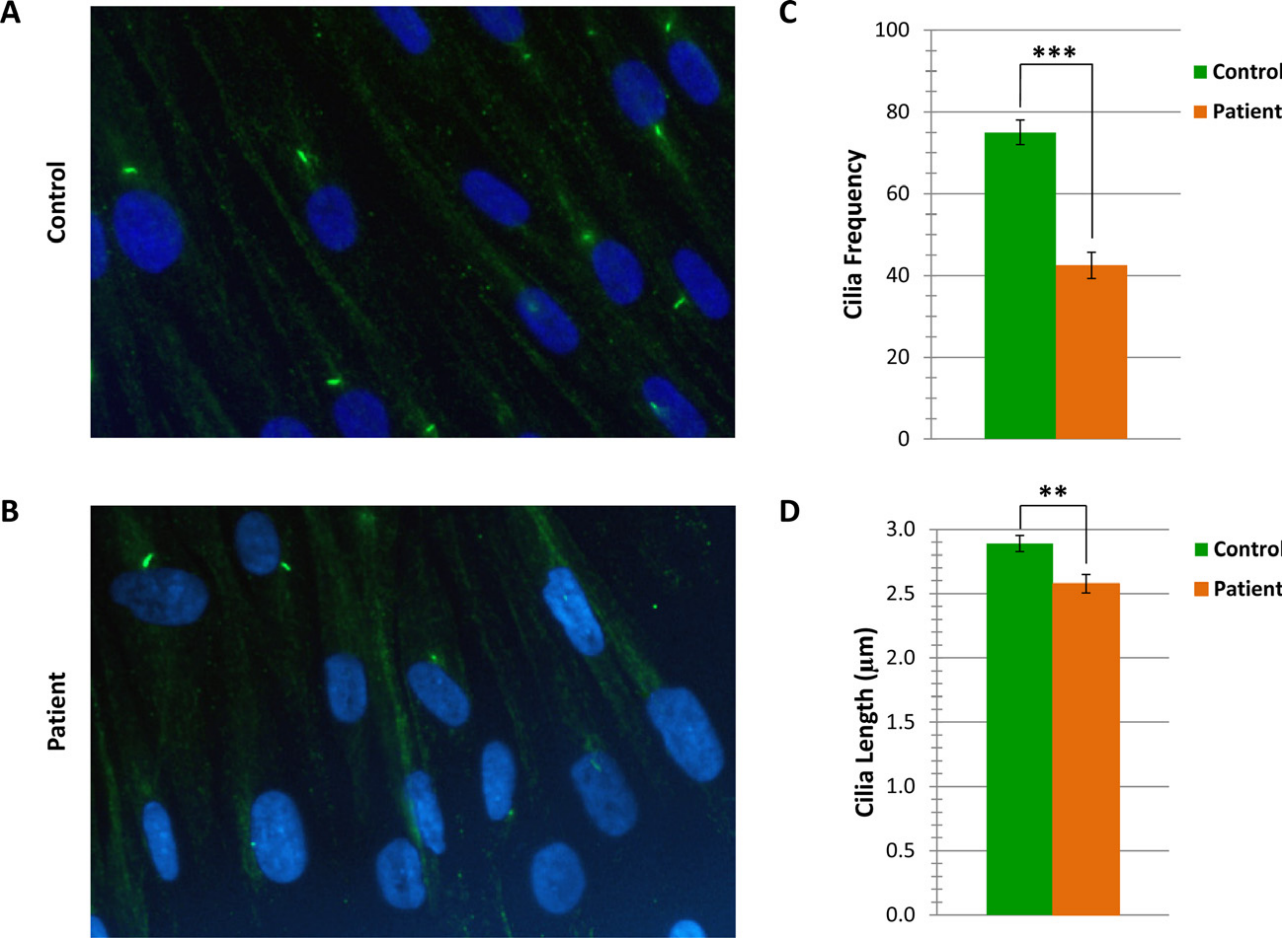
Primary cilia in patient cells exhibit reduced frequency and length as compared with control cells. Primary cilia from serum-starved primary control (A) and patient (B) fibroblasts, stained with anti-acetylated tubulin (green) and counterstained with 4',6-diamidino-2-phenylindole (blue). (C) Patient cells show significantly decreased ciliogenesis versus control cells (*P* < 0.0001, Fisher's exact test). All cells within a total of six fields, representing two independent experiments, were counted for each cell line. Error bars represent the standard error of the mean. (D) Patient cells show significantly decreased ciliary length versus control cells (P < 0.002, unpaired *t*-test). Error bars represent the standard error of the mean.

In order to confirm the pathogenicity of this mutation, we cultured fibroblasts from the foreskin of the younger brother following circumcision and proceeded with stress-induced ciliogenesis assay, essentially as described before (Shaheen et al. 2012). In addition to observing a marked reduction in ciliated fibroblasts, existing cilia in patient fibroblasts were also smaller compared to control age-matched fibroblasts (Fig. 3A and B). These reductions in ciliary frequency and length were confirmed to be highly significant (Fig. 3C and D). Thus, it appears that G623V is associated with a similar ciliogenesis defect to the ones reported in the original description of *IFT122* as a novel CED disease gene (Walczak-Sztulpa et al. 2010). The molecular confirmation of CED in this expands the allelic heterogeneity of *IFT122* in CED (Tsurusaki et al. 2013). In addition, the remarkable cutis laxa phenotype in this family supports one previous report of CED with cutis laxa (Fry et al. 2009) thus confirming that this is a bona fide phenotypic aspect of the disease albeit at low frequency. Finally, this is the first instance of confirmed intellectual disability in CED, which suggests that intellectual disability may be a low-frequency feature of this disorder.
